# Using cyproterone acetate to treat recurrent ischemic priapism in a patient with sickle cell anemia as a comorbidity: a case report

**DOI:** 10.1186/s13256-020-02527-1

**Published:** 2020-10-21

**Authors:** Ali Alshahrani

**Affiliations:** Clinical Pharmacy, Pharmacy College, Taif University, P.O. Box 888, Haweiah, 21974 Saudi Arabia

**Keywords:** Recurrent, Ischemic, Priapism, Sickle cell anemia, Cyproterone acetate

## Abstract

**Introduction:**

The management of recurrent ischemic priapism is unclear in contemporary practice. Yet, if left untreated, the condition may evolve into an acute ischemic priapism and in some cases result in erectile dysfunction. This report documents the results of successful management of recurrent ischemic priapism using cyproterone acetate in a 30-year-old Saudi man with sickle cell anemia as a comorbidity.

**Case presentation:**

A 30-year-old Saudi man denoted visited the emergency room with a painful erection which had lasted for more than four hours. The patient has sickle cell anemia and a family history of sickle cell disease. He is married and has two children. His first priapism case occurred when he was 7 years old. At the age of 15, the condition recurred, and the patient’s doctor prescribed cyproterone acetate 50 mg twice daily for 5 days. The doctor had told him that whenever he was experiencing priapism, he should adhere to this regimen for 5 days. The doctor could not find any guidelines for the prescription of cyproterone acetate.

**Conclusion:**

Priapism cases represent a significant challenge in therapeutic management because of the elevated risk of structural damage to the penis. The fact that there lacks a clinically approved standard approach to managing the condition make it difficult for physicians to effectively manage the condition. Management of the condition is further complicated by existence of comorbidities such as sickle cell anemia. This patient’s case demonstrates that cyproterone acetate prescription is a great preventative strategy that limits priapism recurrences.

## Background

Priapism is an erection physiology disorder characterized by disturbances of the regulatory mechanism governing penile tumescence [1]. Patients with priapism report prolonged penile erection that is not associated with sexual desire [2]. Priapism can either be ischemic, stuttering, or nonischemic. Recurrent ischemic priapism is characterized by self-limiting, prolonged, episodic, sleep-related, and painful erection that lasts for more than 3 hours. The true incidence of this condition is largely undocumented. However, some researchers have pointed out that the annual incidence ranges between 0.5 and 1 cases per 100,000 [3]. Although it is a rare disorder, the condition is known to affect the sickle cell disease (SCD) population more commonly than any other population in the world [4]. There is a reported bimodal peak of the condition at 5–10 years and 20–50 years [5]. In the current literature regarding management of the condition, a clear-cut approach to its treatment is lacking. Both the European Urological Association and the American Urological Association guidelines recognize the existence of the various management options and appreciate the fact that no treatment choice has been established yet. This report contributes to contemporary literature on recurrent ischemic priapism by documenting the results of successful management of the condition using cyproterone acetate in a 30-year-old Saudi man with sickle cell anemia as a comorbidity.

## Case presentation

A 30-year-old Saudi man visited the emergency room with an erection that had lasted more than 4 hours. The erection was painful, and preliminary examination established that he was experiencing ischemic priapism. Moreover, the patient described having a history of priapism dating back to the age of 7.

### Diagnosis

The diagnosis in this case was based on physical examination and patient history. The patient reported severe pain and persistent erection. The patient was already aware of the condition and reported having last experienced the condition at the age of 26 years 4 years ago. The patient acknowledged having received aspiration and irrigation the first time he experienced the condition, followed by the use of different approaches to control the condition. When diagnosing priapism, physical observation of an unintentionally erect penis for a disproportionate duration is the most direct basis for the diagnosis. Typically, a diagnosis process will proceed with the acquisition of patient clinical history, physical examination, and consideration of other characteristics that define the clinical presentation of the condition [6]. Based on the results of the diagnosis, a treatment plan is often developed. When making a diagnosis, the practitioner is concerned with differentiating between ischemic and nonischemic priapism [7]. This is because the former represents a urological emergency, whereas the latter does not. In some cases, radiological and laboratory examinations may be conducted. In this patient’s case, no radiological or laboratory tests were conducted.

The clinical history of the patient and the physical examination of the penis are vital in delineating the features of the condition’s presentation. Practitioners should inquire about the presence of pain, duration of the condition, prior episodes, use and observed success of the used pain-relieving maneuvers, existing causal conditions, and the clinical treatment that was previously used [8]. The clinical practitioner should also establish whether the patient has been using any erotogenic therapies from prescription or nonprescription sources. It is important to establish the erectile function status of the patient before priapism episodes [9]. The presence of pain is indicative of ischemic priapism, and physical inspection may involve palpation of the penis to get information about the extent of rigidity. In nonischemic priapism cases, the presentation is characterized by a partially erect, tumescent, nontender corpora cavernosa. Perineal, abdominal, and rectal examinations may be vital in determining signs of trauma [10]. When laboratory testing has been incorporated into the diagnosis, a number of procedures are conducted, including a complete blood count to determine whether there is an acute infection or hematologic abnormality present in the body [11]. The presence of sickle cells in cases of priapism may be symbolized by the reticulocyte count and hemoglobin electrophoresis [12]. Such tests are a standard practice recommended to all male patients who attend clinics with the presentation of priapism. However, when the priapism is obviously acknowledged as in this patient’s case, the focus shifts to developing a treatment plan for the patient.

### Treatment

Typically, the treatment approaches for priapism are based on the diagnostic category and differ depending on whether the condition is ischemic or nonischemic. In most cases, ischemic priapism is managed through the elimination of the ischemic effects of the compartment syndrome that are present in the penis [13]. For major episodes as in this patient’s case, initial intervention often tends to involve penile aspiration, which may be combined with the drainage of blood. These approaches serve as a vital first step in relieving pain and counteracting local acidotic and anoxic metabolic derangements [14]. The practitioner at this point can take samples for cavernous blood gas testing. When the patient was 7 years old, he had gone to the emergency room many times regarding priapism, especially in the night, and his blood had a combination of aspiration and irrigation from around the penis, then was discharged [15]. The recurrence of the condition at the age of 26 received a different management approach because the managing doctor first advised him to take walks and go up and down the stairs. After the condition persisted, the doctor prescribed sildenafil citrate, a prescription that would then be changed to cyproterone acetate 50 mg twice daily.

For the management of the condition in this patient, first-line therapy was invoked whereby penile aspiration in combination with an extracavernous α-agonist injection was conducted. A preceding dorsal nerve block was performed for anesthetic purposes. Once the patient’s condition stabilized, a dose of cyproterone acetate 50 mg daily for the next three weeks was recommended. The recommended dosage was based on existing literature on the treatment of ischemic priapism in the general population [16].

### Outcome and follow-up

The patient was immediately stabilized, and the use of a tablet of cyproterone acetate 50 mg daily for 1 week was recommended. Follow-up was done after 1 week, when the patient reported that the condition had subsided. He was then asked to reduce the dosage to one tablet per week. The next follow-up was done after a two months, where the patient stated that the condition only reappeared after he stopped using the medication 3 weeks after consistent use of the medication for the first month. However, after going through the dosage consistently for 1 week, the condition disappeared. It has been 1 year since the condition was last reported. It is worth mentioning that the use of the medication has not affected the patient’s sexual activity; he reported having sex twice per week. However, the patient admits that he is always afraid that the condition may recur.

## Discussion

Ischemic priapism is a delicate condition that occurs commonly among people with SCD. Studies estimate that only 44% of those who experience ischemic priapism for 24–36 hours recover from the risk of erectile function [17]. As such, time is a critical factor in the management of the condition. The primary goal of any medical practitioner is to decompress the corporal bodies and reestablish arterial blood flow in order to reduce the risk of tissue necrosis, the ameliorating pain, and injury. Contemporary practice in ischemic priapism management recommends a progressive approach to the condition that should be completed in a stepwise fashion in order to achieve a prompt resolution [18].

The underlying mechanical factor in ischemic priapism is the sludging of erythrocytes leading to veno-occlusive disease in the sinusoids of the corpus cavernous. Molecular studies have established that the condition results from a tonically deficient endothelial nitric oxide (NO) synthesis by the cavernous smooth muscle endothelium [19]. The deficiency orchestrates a downregulation of cyclic guanosine monophosphate (cGMP)-specific protein kinase [20]. It also results in lowered levels of phosphodiesterase-5 (PDE5). During sexual stimulation, psychogenic, reflexogenic, or nocturnal, there is no source of cGMP and NO that overwhelms the reduced levels of PDE5 set point, giving rise to the prolonged erection [21]. Researchers have also noted an accumulation of adenosine under hypoxic conditions, further increasing cyclic adenosine monophosphate and protein kinase A activation, resulting in low intracellular calcium and relaxation [22]. Through the endothelial cells, adenosine is known to increase NO release, thus resulting in a synergistic effect that contributes to ischemic priapism [22, 23]. In recurrent ischemic priapism mouse models with SCD, researchers have found high levels of adenosine compared with the control group. More recent studies have also established a novel role of opiorphins, a family of peptides, in the regulation of penile smooth muscle tone and development of priapism. Overexpression of the genes encoding opiorphins is thought to increase corporal smooth muscle relaxation, priapism outcomes, and enhanced erectile function [24].

The goal of managing recurrent ischemic priapism is to prevent future episodes. Success of therapies is defined by reduced recurrence of the condition and no side effects such as loss of libido, erectile dysfunction, gynecomastia, and hot flashes [21]. For the present patient’s case, success was defined on the basis of patient satisfaction, nonrecurrence of the condition, and lack of side effects. The patient reported that the management strategy adopted was the best compared with his other experience. It has been 2 years since the last episode was reported, and the patient reports having a normal sex life (two or three times per week). The European Urological Association and the American Urological Association guidelines recognize the role that preventive measures in the management of ischemic priapism have in preventing fibrotic transformation of corpora cavernosa and erectile dysfunction [25]. In addition, these association guidelines recognize the existence of the various management options available for the condition but appreciate the fact that no treatment choice has been established yet [26]. The limited information available in the literature supporting levels III and IV evidence derives from the small number of control studies and has been the primary reason for lack of an established choice of treatment. However, various animal and human studies have shown that androgens play a vital role in the regulation of ischemic priapism [27]. The use of cyproterone in this case was based on its established properties in research. Cyproterone acetate belongs to a class of drugs that alter the hormonal environment within the male erectile system [23, 28]. Specifically, cyproterone acetate works by inhibiting androgen receptors. Androgen is known for maintaining penile tissue and erectile nerve integrity during an erection. Androgen is also known for modulating the central and peripherical mechanisms during erectile function. By inhibiting androgen receptors, cyproterone acetate is able to achieve penile tissue trophy [23, 29, 30]. Furthermore, deprivation of androgen is known to result in alteration in dorsal nerve structure and endothelial morphological changes in smooth muscle, collagen content, and fat and to diminish protein expression. Imperatively, the elimination half-life of the oral cyproterone acetate is 1.6–4.3 days.

## Conclusion

This case demonstrates that cyproterone acetate prescription is a great preventative strategy that limits ischemic priapism recurrence among patients with SCD as a comorbidity. This is because the medication’s progestogenic action exerts a negative effect on the hypothalamic receptors, thus diminishing the production of testicular androgens. The key to successful management of recurrent ischemic priapism is acknowledgement of the ischemic feature of the condition’s clinical presentation.

### Learning points

- The management of priapism, in general, is practically applied on the basis of diagnostic category.
- This is a classic case of a rare penile complication, and even though recurrent ischemic priapism is considered distinct from major nonischemic priapism, it calls for management practices that acknowledge the ischemic feature of its clinical presentation.
- As demonstrated in this case, cyproterone acetate prescription is a great preventative strategy that limits priapism recurrence.
- Imperatively, priapism holds a special place in contemporary sexual medicine. The risk of complete loss of erectile function means that clinical practitioners should exercise care when offering clinical management of the condition.

## Data Availability

All data in this study are included in this case report.
